# Brosimine B and the biphasic dose-response: insights into hormesis and retinal neuroprotection

**DOI:** 10.3389/fphar.2025.1558726

**Published:** 2025-04-08

**Authors:** Susanne Suely Santos Fonseca, Natacha M. S. Port’s, Gisele Priscila Soares Aguiar, Eliã P. Botelho, Nádia M. G. Couto, Wandson Braamcamp Souza Pinheiro, André Salim Khayat, Elizabeth S. Yamada, Edmar T. Costa, Chubert Bernardo C. Sena, Mara Silvia P. Arruda, Carlomagno P. Bahia, Antonio Pereira

**Affiliations:** ^1^ Institute of Technology, Federal University of Pará, Belém, Pará, Brazil; ^2^ Oncology Research Center, Hospital University João of Barros Barreto, Federal University of Pará, Belém, Pará, Brazil; ^3^ Laboratory of Neuroplasticity, Institute of Health Sciences, Federal University of Pará, Belém, Pará, Brazil; ^4^ Central Extraction Laboratory, Federal University of Pará, Belém, Pará, Brazil; ^5^ Laboratory of Experimental Neuropathology, Hospital University João of Barros Barreto, Federal University of Pará, Belém, Pará, Brazil; ^6^ Laboratory of Structural Biology, Federal University of Pará, Belém, Pará, Brazil

**Keywords:** oxidative stress, Brosimine b, retinal cell, neuroprotection, hormesis, natural products, antioxidant

## Abstract

**Introduction:**

The biphasic dose-response behavior, also known as hormesis, is a characteristic feature of numerous natural products. It is defined by beneficial effects at low concentrations and toxicity at higher doses. This study investigates the hormetic effects of Brosimine B, a flavonoid derived from Brosimum acutifolium, on retinal cell viability under oxidative stress.

**Methods:**

To simulate ischemic conditions, we used an oxygen-glucose deprivation (OGD) model. Retinal cells were treated with varying concentrations of Brosimine B, and analyses of cell viability, reactive oxygen species (ROS) production, and antioxidant enzyme activity were performed.

**Results:**

Brosimine B at 10 µM significantly enhanced cell viability and reduced ROS production, likely through modulation of oxidative stress-protective enzymes such as catalase. However, higher concentrations (>10 µM) induced cytotoxic effects. A computational modeling approach using a hormetic (inverted U-shaped) model revealed biologically interpretable parameters, including a peak response at 10.2 µM and a hormetic zone width (σ = 6.5 µM) (R^2^ = 0.984).

**Discussion:**

These results confirm that Brosimine B exhibits hormetic neuroprotective effects within a well-defined concentration window, supporting its potential as a therapeutic agent for oxidative stress–related retinal damage. The study highlights the value of computational modeling in optimizing dose–response analyses, offering a framework for refining natural product therapies and predicting toxicological thresholds in pharmacological applications.

## 1 Introduction

The Moraceae family is prevalent in tropical and subtropical regions, with species that can grow up to 40 m tall in the Amazon rainforest. Within this family, the Brosimum genus includes *Brosimum acutifolium* (commonly known as Mururé), a tree valued for its medicinal, nutritional, and economic importance. Its bark and latex are traditionally used for their analgesic and anti-inflammatory properties, while its wood is a vital resource for the timber industry (Torres et al., 2000; Takashima and Ohsaki, 2001). Beyond its utilitarian significance, *B. acutifolium* is also a reservoir of bioactive compounds, including flavonoids and terpenes, which exhibit potent antioxidant (Martins et al., 2016) and antibiofilm activities against *Staphylococcus aureus* (Reis et al., 2020). One of its secondary metabolites, Brosimine B, has shown promising anticancer effects, particularly against glioblastoma (Maués et al., 2019; Garcês de Couto et al., 2020) and chronic myeloid leukemia cell lines (Willig et al., 2023), highlighting its potential for therapeutic development.

Hormesis, which describes a biphasic dose-response in which low doses of a compound can elicit beneficial effects while higher doses become toxic, provides a valuable framework for studying natural products such as Brosimine B (Mattson, 2008; Nascarella and Calabrese, 2012). While many naturally derived compounds display this dose-dependent duality, the hormetic properties of most remain poorly understood. By investigating how Brosimine B from Brosimum acutifolium behaves across different concentrations, researchers can uncover both its protective mechanisms and potential adverse effects. Such insights not only guide therapeutic applications but also support the responsible and sustainable use of Amazonian biodiversity.

Oxidative stress, arising from an imbalance between reactive oxygen species (ROS) production and antioxidant defenses, plays a pivotal role in cellular adaptation, survival, and pathology (Jaganjac et al., 2022). At moderate levels, oxidative stress activates protective pathways, enhancing cellular resilience in a phenomenon linked to hormesis (Nitti et al., 2022; Bondy, 2023). However, excessive ROS levels can lead to damage associated with diseases such as cancer (Jelic et al., 2021), metabolic disorders (Incalza et al., 2018), and circulatory dysfunctions (Rubattu et al., 2019).

The retina is particularly susceptible to oxidative stress due to its high metabolic activity and constant exposure to light (Nebbioso et al., 2022). Prolonged oxidative stress can compromise retinal structure and function, triggering degenerative processes that impair vision (Masuda et al., 2017). The avian retina, which shares its embryonic origin with the central nervous system (CNS), is an excellent model for investigating oxidative stress and neuroprotection. Its structural and functional parallels with the CNS—such as similar neuron and glial cell types, neurotransmitter systems, and protective signaling pathways—provide a powerful window into broader CNS mechanisms. Because these cellular processes are highly conserved across species, studying how the avian retina responds to and mitigates damage under stress conditions may offer valuable insights into neuroprotective strategies in mammalians as well (Osborne and Neuhoff, 1983; Belecky-Adams et al., 2008; Doh et al., 2010).

This study investigates the hormetic effects of Brosimine B on retinal cell cultures exposed to oxygen-glucose deprivation (OGD), an *in vitro* model of ischemia-induced oxidative stress (Park et al., 2023). We also modeled Brosimine B’s biphasic dose-response dynamics, enabling precise characterization of its stimulatory and toxic thresholds. By integrating experimental and computational approaches, this work provides valuable insights into Brosimine B’s therapeutic potential and its role in mitigating oxidative stress, laying the groundwork for future pharmacological applications.

## 2 Materials and methods

### 2.1 Plant material

The trunk bark of *B. acutifolium* subs. acutifolium was collected in the Moju municipality, located 266 km from Belém-Pará (PA), Brazil. A fertile sample was identified and registered with the herbarium of the Brazilian Agricultural Research Corporation (EMBRAPA Amazônia Oriental) under the number IAN-187036. The collected species was also registered in the Brazilian National System for Genetic Heritage Management and Associated Traditional Knowledge, with the registration number A678D8C.

### 2.2 Extraction and isolation

**FIGURE 1:**
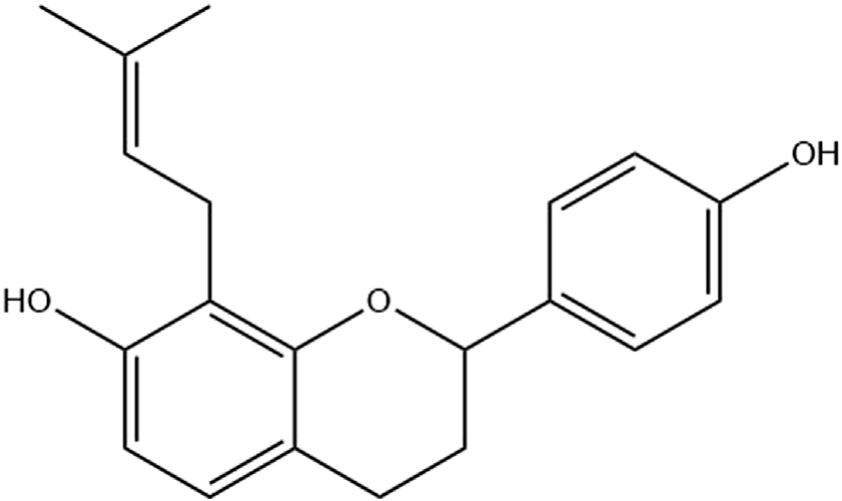
Chemical structure of Brosimine B.

The Brosimine B compound (4,7-dihydroxy-8(3,3-dimethylallyl)flavan) (Figure 1) was isolated from ethanolic extract (1:10 m/v proportion) from the *B. acutifolium* bark. The powdered bark was submitted to 48 h of maceration in a percolating system, using ethanol 96° GL as extraction solvent, renovated every 24 h. Subsequently, the extract was concentrated at reduced pressure and dried. Then, it was subjected to fractionation by silica gel column chromatography, using a gradient elution of hexane: ethyl acetate (9.5:0.5 to 65:35). The samples obtained were monitored by Thin Layer Chromatography to verify the isolation of the substance. The structure of Brosimine B was identified by 1H and 13C NMR spectra and compared with the literature. Spectral data were acquired at 300 and 75 MHz, respectively, on a Varian GEMINI 300 instrument (Garcês de Couto et al., 2020; Torres et al., 2000; Figure 1).

### 2.3 Mixed retinal cell culture

Primary cultures of retinal cells from 7-day-old chicken embryos were prepared from fertilized eggs of *Gallus gallus domesticus*, following approval from the Ethical Committee for the Use of Animals (CEUA) of the Federal University of Pará (authorization #19314). The retinae were carefully dissected in a sterile environment using a calcium and magnesium-free salt solution (128 mM NaCl, 4 mM KCl, 1 mM Na2HPO4.H2O, and 12 mM glucose) and then cultured for 7 days in Dulbecco’s Modified Eagle Medium (DMEM) containing 10% fetal bovine serum (FBS), 2 mM glutamine, and 2% penicillin-streptomycin.

### 2.4 Oxygen and glucose deprivation and compound treatment

Hypoxia was induced *in vitro* using oxygen and glucose deprivation (OGD) (Chu et al., 2008). The cells were incubated in a CO_2_ chamber in sealed plates for 3, 6, or 24 h and cultured in DMEM medium without FBS and containing a low concentration of glucose (5.5 mM), as well as 1% non-essential amino acids and antibiotics (P/S). Control cells were incubated in unsealed plates containing DMEM medium with 25 mM glucose and 10% FBS.

The effects of Brosimine B were evaluated in control cells by administering the compound at concentrations of 1, 5, 10, 25, 50, and 100 µM for 24 h to non-OGD retinal cell cultures. To evaluate the protective effects of Brosimine B, the compound was administered at a concentration of 10 µM in a 0.06% DMSO solution to OGD cell cultures at the beginning of the hypoxic period for 3, 6, or 24 h.

### 2.5 Cell viability

We evaluated cell viability using the 3-(4,5-dimethylthiazolyl-2)-2,5-diphenyltetrazolium bromide (MTT) (Sigma-Aldrich) colorimetric assay (Jiang et al., 2015). Briefly, cells were washed with phosphate-buffered saline (PBS) and incubated with 200 µL of MTT (0.5 mg/mL) for 3 h (Jiang et al., 2015). After, cells were homogenized with dimethyl sulfoxide (DMSO) 99.9% to solubilize formazan crystals. The colorimetric analysis was performed in a 96-well microplate reader (Elx800/Biotek) with a 570 nm wavelength. The reference was 150 µL of DMSO. Cell viability was expressed as a percentage of control.

### 2.6 Biphasic dose-response curves

To characterize the biphasic dose-response behavior of Brosimine B, we employed a modeling approach using experimental data. The response was measured as cell viability under varying concentrations of Brosimine B (1, 5, 10, 25, 50, and 100 µM). We implemented a hormetic model based on an inverted U-shaped Gaussian function:

$$Y=baseline+amplitude.\;e^{-\frac{{\left(x-x_{0}\right)}^{2}}{{2\sigma }^{2}}}$$

where baseline represents the control response, amplitude captures the peak above baseline, **x**_**0**_ defines the concentration at which maximal stimulation occurs, and σ determines the width of the hormetic response.

The model was fitted using non-linear least squares, and its performance was evaluated using R^2^ and RMSE (Root Mean Square Error) to compare goodness-of-fit.

### 2.7 Quantification of intracellular superoxide anions

The cellular production of superoxide anions was determined using the Nitro Blue Tetrazolium Chloride (NBT) assay (Choi et al., 2006; Fonseca et al., 2024). Cell cultures were subjected to different periods of hypoxia (3, 6, or 24 h) and treated with Brosimine B (10 µM). They were then washed with PBS and incubated in either low glucose DMEM (5 mM) without FBS (OGD cultures) or high glucose DMEM (25 mM) with 10% FBS (non-OGD cultures) containing NBT (0.2 mg/mL) in a CO_2_ incubator for 2 h. The cells were then rinsed with PBS, fixed with methanol, and solubilized using 2 M potassium hydroxide. The formazan crystals were solubilized in 99.5% DMSO for quantification with a 96-well microplate reader (El x 800/Biotek) at a wavelength of 620 nm.

### 2.8 Catalase activity

Cell cultures were washed twice with PBS at 4°C, and the intracellular content was obtained after ultrasonic exposure for 10 seconds in PBS. Samples were centrifuged using an MPW-350/MPW centrifuge at 14,000 G for 10 min at 4°C. The resulting supernatant was then carefully collected for enzyme activity assays and protein quantification. We used an indirect method to quantify catalase activity based on the peroxidation activity of this enzyme while reacting with hydrogen peroxide (H_2_O_2_) in an alcoholic medium to produce aldehyde and water. The aldehyde reacts with the chromogen 4-Amino-3-hydrazino-5-mercapto-1,2,4-triazole (Purpald^®^) and forms a bicyclic heterocycle with the aldehyde, which, upon oxidation, changes from colorless to purple color with an intensity proportional to the amount of aldehyde and which can be determined with a spectrophotometer at 540 nm. We used different concentrations of formaldehyde (5–75 mM) as standard curves. The results of the catalase enzyme assay were normalized to the total protein content present in the samples and expressed as µg/mg protein.

### 2.9 Protein quantification

The protein content was determined using the Bradford method (Bradford, 1976). A Coomassie Brilliant Blue G-250 (0.1 mg/mL) solution was prepared by mixing the dye with ethanol (5%) and phosphoric acid (8.5%). 5 μL of the sample was then added to this solution, and the absorbance was measured with a microplate reader after a 2-min incubation period. Bovine serum albumin (BSA) was used as a comparative standard, with known concentrations of 200, 250, 375, 500, and 750 µg/mL. The samples were analyzed using spectrophotometry with the detection wavelength set at 595 nm.

### 2.10 Statistical analysis

The descriptive results were expressed as the mean ± standard deviation. Statistical significance was set at 0.05, and differences between groups were evaluated using a one-way or two-way analysis of variance (ANOVA), followed by Tukey’s or Bonferroni’s *post hoc* test. Each assay was performed in three independent experiments, with each experiment conducted in triplicate.

## 3 Results

### 3.1 Structural identification of Brosimine B

Spectral data of Brosimine B is in accordance with the literature (Torres et al., 2000; Garcês de Couto et al., 2020). The compound is identified as 2(S)-7,4′-dihydroxy-8-(3″,3″- dimethylallyl)flavan: White crystal, C_20_H_22_O_3_. The ^1^H NMR spectrum (300 MHz, CDCl_3_) displayed the following resonances: δ_H_ 4.98 (dd, J = 10.5, 2.1 Hz, H-2), 2.15 (m, H-3eq), 1.96 (m, H-3ax), 2.72 (ddd, J = 16.0, 8.7, 1.8 Hz, H-4eq), 2.92 (ddd, J = 16.2, 11.7, 5.7 Hz, H-4ax), 6.82 (d, J = 8.2 Hz, H-5), 6.41 (d, J = 8.4 Hz, H-6), 7.29 (d, J = 8.6 Hz, 2H, H-2’/6′), 6.84 (d, J = 8.6 Hz, 2H, H-3’/5′), 3.42 (d, J = 10.2 Hz, 2H, H-1″), 5.27 (m, H-2″), 1.74 (s, 3H, Me-cis), 1.72 (s, 3H, Me-trans) (Supplementary Figure S1). The ^13^C NMR spectrum (75 MHz, CDCl_3_): revealed signals at δ_C_ 155.0 (C-4′), 153.5 (C-7), 152.9 (C-9), 134.4 (C-1′), 134.0 (C-3″), 127.3 (C-5), 127.3 (C-2’/6′), 122.2 (C-2″), 115.2 (C-3’/5′), 114.5 (C-8), 113.9 (C-10), 108.1 (C-6), 77.4 (C-2), 30.0 (C-3), 25.8 (Me-trans), 24.9 (C-4), 22.4 (1″), 17.8 (Me-cis) (Supplementary Figure S2).

### 3.2 Brosimine B and cell viability

**FIGURE 2:**
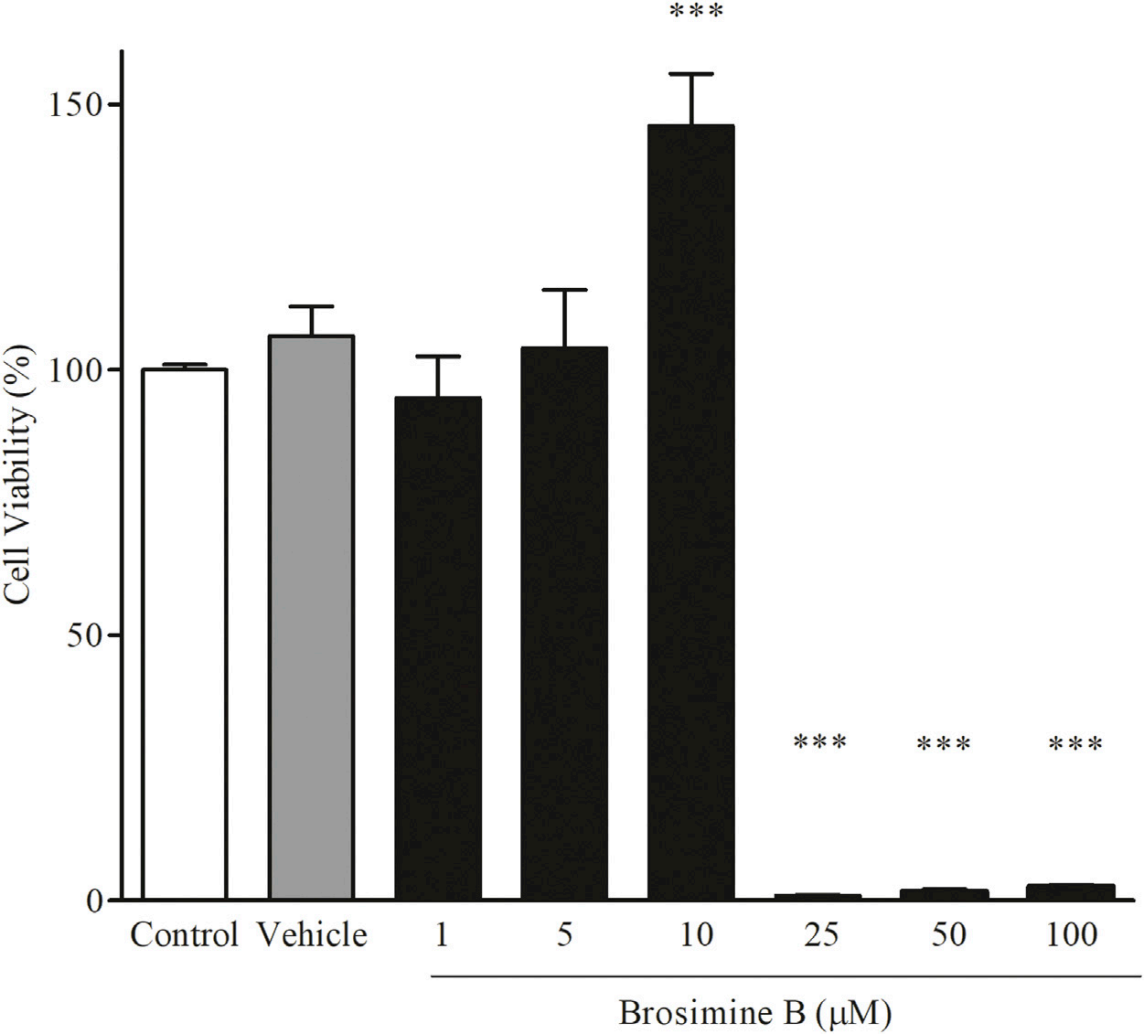
Effect of Brosimine B on cell viability after 24 h in culture. Retinal cells in normal culture were treated with different concentrations of Brosimine B (1, 5, 10, 25, 50, and 100 µM) for 24 h. Cell viability was analyzed with the MTT assay. All values are normalized to control and expressed in percentage. ^***^p < 0.0001.

We analyzed cell viability under different concentrations of Brosimine B (1.00, 5.00, 10.00, 25.00, 50.00, and 100.00 µM) in mixed retinal cell cultures. We observed a significant increase in cell viability (relative to the control group) only when Brosimine B reached a concentration of 10 μM (145.0% ± 9.80%, p < 0.0001; F = 350.00), but not 1 μM (94% ± 7.8% p < 0.0001; F = 350.00) or 5 μM (104% ± 10.9%, F = 350.00). Exposure of the mixed culture to higher concentrations of Brosimine B drastically reduced cell viability: 25.00 μM (0.95 ± 0.15, **p < 0.0001, F = 350.00), 50.00 μM (1.72 ± 0.20, *p < 0.0001, F = 350.00), and 100.00 μM (2.64 ± 0.18, *p < 0.0001, F = 350.00) (Figure 2).

### 3.3 Biphasic dose-response curves

**FIGURE 3:**
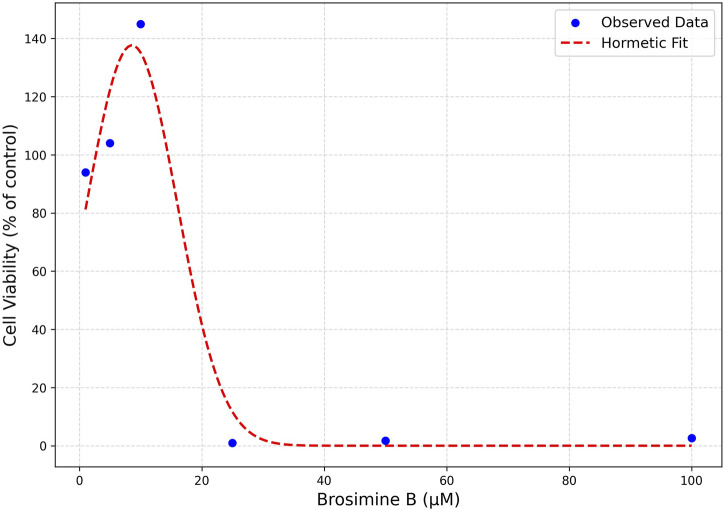
Hormetic Model Fit of Brosimine B Dose-Response Curve. Cell viability is expressed as a percentage relative to the control. Observed data points (blue dots) represent measured cell viability at various Brosimine B concentrations. The fitted parameters were baseline = 0.715, amplitude = 0.217, x_0_ (peak concentration) = 10.2 µM, and σ (spread) = 6.5 µM.

Brosimine B’s biphasic dose-response was effectively captured by a hormetic (inverted U-shaped) Gaussian function, providing a strong fit to the experimental data $\left(R^{2}=0.984;\;RMSE=0.076\right)$ (Figure 3). The fitted parameters were baseline = 0.715, amplitude = 0.217, x_0_ (peak concentration) = 10.2 µM, and σ (spread) = 6.5 µM. This model reflects the stimulatory effect of Brosimine B at lower concentrations, peaking around 10 μM, followed by cytotoxic responses at higher doses, aligning with hormesis commonly observed in natural products.

### 3.4 Protective effect of Brosimine B during hypoxia

**FIGURE 4:**
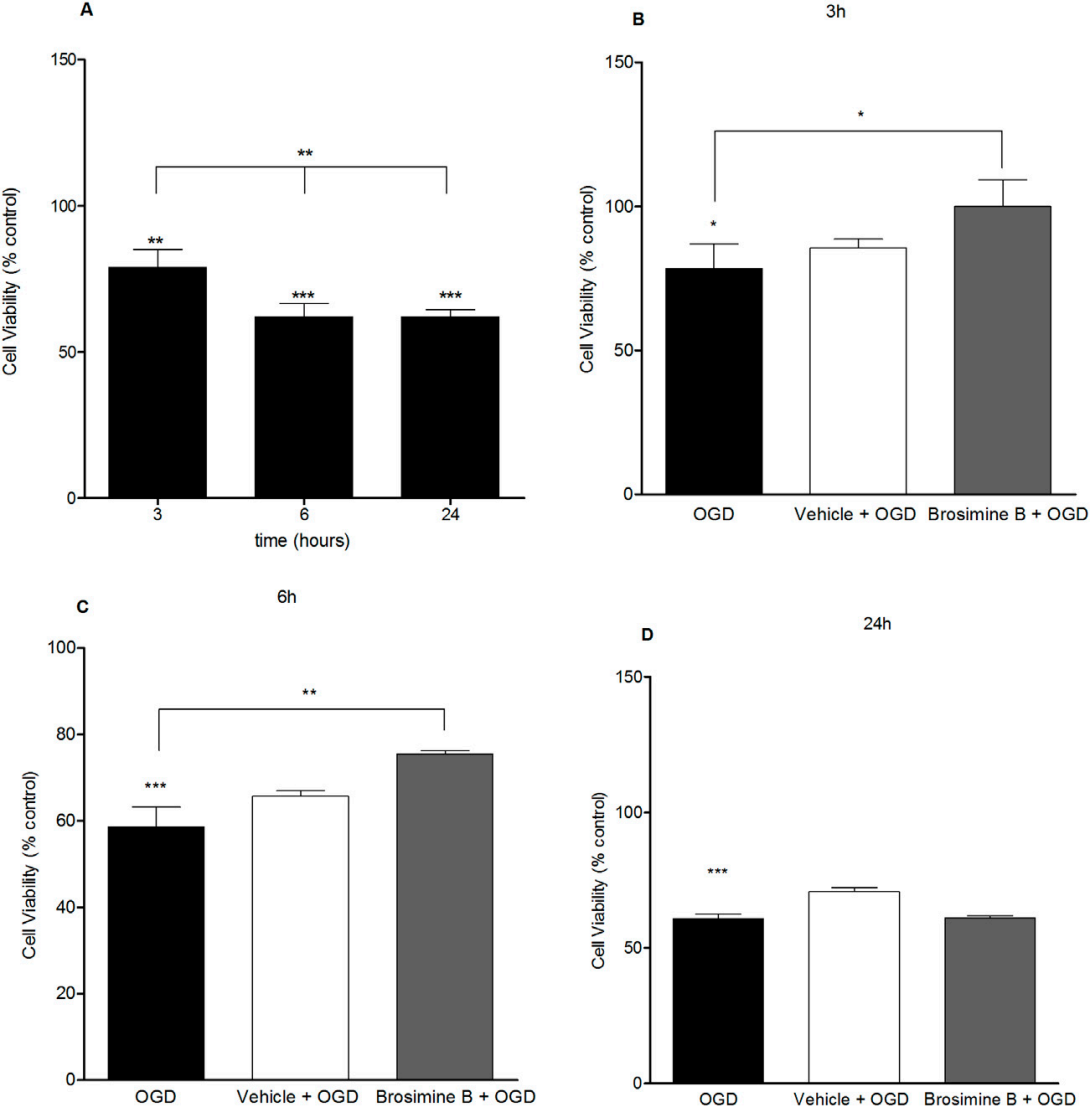
Protective effect of Brosimine B on embryonic retinal cells subjected to OGD. OGD condition reduces cell viability at 3, 6, and 24 hours **(A)**. Brosimine B exerts a protective effect on embryonic retinal cells exposed to OGD for 3 **(B)**, 6 **(C)**, and 24 hours **(D)**. Results are expressed as percentage relative to control. Statistical significance: ***p < 0.0001 compared to the control; **p < 0.0001 compared to the OGD group.

OGD caused a significant reduction in cell viability in all periods of exposure: 3 h (80.0% ± 2.10%; Interaction:13.00, *p < 0.005), 6 h (58% ± 4.60%; Interaction:13.00, ***p < 0.0001), and 24 h (62% ± 2.50%; Interaction:13.00, ***p < 0.0001), compared to the control group. To evaluate the protective effects of Brosimine B on OGD retinal cell cultures, we chose the concentration that increased cell viability in the previous experiment (10 µM) (Figure 4). After the addition of 10 µM of Brosimine B to OGD cell cultures, cell viability increased after a period of 3 h (107.00% ± 6.20%; ^###^p < 0.0001; Interaction:13.00) and 6 h (25.00% ± 0.70%; ^##^p < 0.001; Interaction:13.00), but not 24 h (39.00% ± 1.40%; ns; Interaction:13.00) (Figure 4).

### 3.5 Oxidative stress and Brosimine B

**FIGURE 5:**
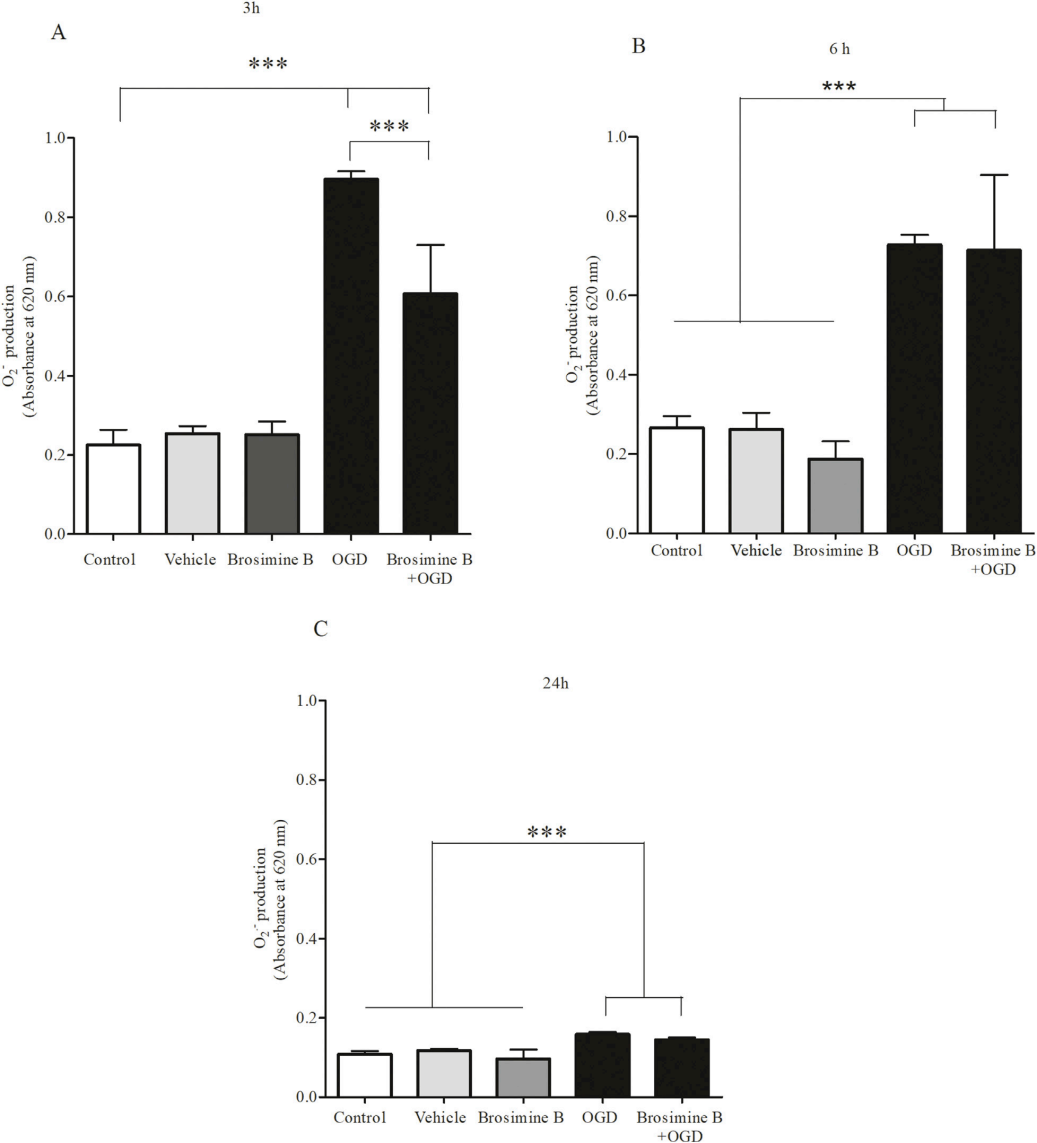
Effect of Brosimine B on ROS Production in Cells Subjected to OGD. Brosimine B (10 μM) reduces ROS production in cell cultures exposed to OGD for 3 h **(A)**, 6 h **(B)**, and 24 h **(C)**. Results are expressed relative to the control. Statistical significance: ***p < 0.0001 compared to control and ^###^p < 0.0001 compared to the OGD group.

There was a significant increase in ROS production after 3 h (313.00% ± 8.80%; ***p < 0.0001), 6 (453.00% ± 27.00%; Interaction 19.64, ***p < 0.0001), and 24 h (208.00% ± 6.70%; Interaction 19.64, **p < 0.005) of OGD (Figure 5). Administration of 10 µM of Brosimine B to retinal cell cultures submitted to OGD decreased ROS levels, but only in the 3 h period after induction of hypoxia (269.00% ± 54.00%; Interaction 19.64, ^###^p < 0.0001) (Figure 5A), with no effect being observed for the 6 h (426.00% ± 30.00%; Interaction 19.64, ns, p > 0.05) (Figure 5B), and 24 h (210.00% ± 7.90%; Interaction 19.64, ns, p > 0.05) period (Figure 5C).

### 3.6 Catalase activity during hypoxia

**FIGURE 6:**
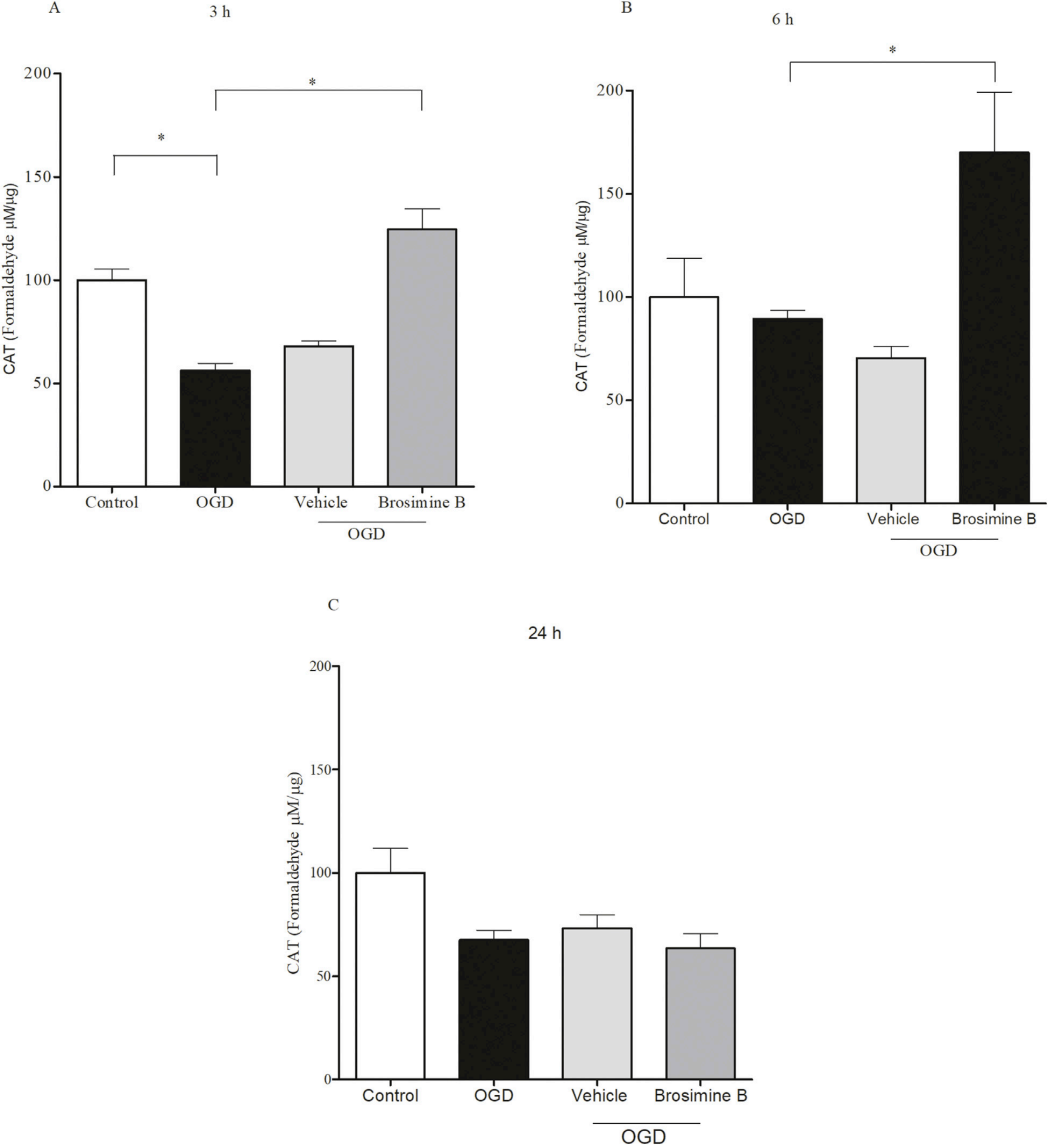
Effect of 10 μM Brosimine B on the activity of the enzyme catalase in retinal cell cultures during OGD for 3, 6 or 24 h *in vitro* **(A–C)**, respectively). The catalase activity was analyzed using an assay de catalase. **p < 0,01, in comparison with a control group and OGD. Error bars represent Mean ± SD; *p < 0.005, comparing with control groups and OGD; ***p < 0.0001 comparing with OGD by one-way analysis of variance (ANOVA), post-test Tukey.

Since the enzyme catalase protects cells from oxidative damage by ROS, we evaluated the effect of Brosimine B on catalase activity during OGD in retinal cell cultures. After 3 h of OGD (Figure 6), catalase activity was significantly reduced (56.20% ± 6.11%; Interaction 26.11,***p < 0.001) and treatment with 10 μM of Brosimine B caused a significant increase in catalase activity (124.00% ± 17.20%; Interaction 26.11, ***p < 0.001) compared to both the control (100.00% ± 9.50%; Interaction 26.11, ***p < 0.001) and OGD (56.00% ± 6.11%; Interaction 26.11, ***p < 0.001) groups (Figure 6A). After 6 h (Figure 6B) of OGD there was no significant difference in catalase activity (89.00% ± 8.20%; *p < 0.05) when compared to controls (100.00% ± 32.00%; Interaction 26.11, *p < 0.05), but treatment with 10 µM of Brosimine B significantly increased enzymatic activity (141.00% ± 10.00%; Interaction 26.11, #p < 0.05) compared to the OGD group. After 24 h of OGD, catalase activity was similarly decreased in both treated (63.30% ± 14.36%; Interaction 26.11, ns,p > 0.05) and untreated groups (67.20% ± 7.85%; Interaction 26.11, ns, p > 0.05), compared to controls (100.00% ± 20.40%; Interaction 26.11, ns, p > 0.05) (Figure 6C).

## 4 Discussion

The *Brosimum acutifol*ium tree has long been used by indigenous people from South America for its medicinal properties (Takashima and Ohsaki, 2001). Extracts from its bark have been found to be rich in flavonoids and terpenes and display intense antioxidant activity (Martins et al., 2016). Since the *B. acutifolium* tree has been continually used for timber in the region, there is a growing worldwide concern about introducing more sustainable practices in that industry. In the present work, we provide results from *in vitro* studies of retinal cell cultures submitted to OGD showing that the flavan Brosimine B, extracted from the *B. acutifolium* tree bark, is a neuroprotectant agent that could be further exploited as a potential therapeutic candidate for fighting oxidative stress in the brain. Retinal cell cultures serve as a valuable model for neuroprotection studies, as they share key physiological and pathological features with CNS neurons, making them particularly relevant for investigating oxidative stress and neurodegenerative mechanisms (Mathew et al., 2020; Reichenbach and Bringmann, 2020; Deppe et al., 2024).

Oxidative stress results from the production of ROS following ischemic brain events (Shaafi et al., 2021). Oxidative stress may overwhelm intrinsic antioxidant neuronal defenses, such as the production of the enzymes glutathione peroxidase, catalase (CAT), and superoxide dismutase (SOD) (Salim, 2017), leading to the expression of caspases that trigger apoptosis in the first hours following ischemia (Benchoua et al., 2001; Radak et al., 2017). One of the main therapeutic goals after stroke, for instance, is to protect cells threatened by oxidative stress in the penumbra zone by using effective antioxidant measures. Our results showing a decrease in retinal cells’ viability due to OGD confirm previous studies describing increased rates of cell apoptosis after just 30 min of OGD *in vitro* (Wise-Faberowski et al., 2001; Liu and McCullough, 2013). The negative impact of OGD on cell viability could stem from cellular events such as excitotoxicity and oxidative stress (Asuni et al., 2015). However, our results also show that cell viability stabilizes after 6 h of OGD (see Figure 4). We hypothesize that in mixed brain cell cultures, neurons and glia react differently to ischemia(Jones et al., 2011; Becerra-Calixto and Cardona-Gómez, 2017). Neurons are more susceptible to ischemic death than glial cells and die more extensively in the first 6 h of the ischemic insult. Some glial cells, however, proliferate in response to ischemia (Sofroniew, 2015; Liu and Chopp, 2016), helping to offset the effect of neuronal death and stabilizing the total number of cells after 6 h of OGD as seen in our study (see Figure 4).

The increase in cell viability we observed with Brosimine B 10 µM in normoxia is also observed with other natural products in other reported *in vitro* essays (Somaida et al., 2020; Fonseca et al., 2024). For instance, another flavonoid, hesperidin, increased the number of neural progenitor cells and post-mitotic neurons by 41% and 21%, respectively, after 24 h of treatment of co-cultures of neurons and astrocytes (Nones et al., 2012). Other isolated plant compounds potentiate the action of neuroprotective mechanisms, as shown in *in vivo* studies with acetonide (verbascoside), a caffeoyl phenylethanoid glycoside that was able to decrease infarct area, brain edema, and ameliorate oxidative stress and neuronal apoptosis following focal cerebral ischemia in rats (Xia et al., 2018).

The neuroprotective effect of Brosimine B we reported could be due to its positive influence on intrinsic cellular mechanisms counteracting the effects of oxidative stress (Maués et al., 2019). The Moraceae plant family, for instance, is well known for having large concentrations of antioxidants that offset the increase of oxidative compounds after ischemic brain injury (Florian et al., 2006; Khan et al., 2013). Compounds isolated from plant species belonging to this family, such as moracin P and Mulberrofuran Q, were able to increase cell viability after 20 h of OGD *in vitro* (Lee et al., 2011). Like our results, these compounds also showed cytotoxic effects *in vitro* in concentrations above 10 µM (Lee et al., 2011).

Flavonoids can counterbalance oxidative stress generated by ischemia and help reduce secondary ischemic injuries and neurological damage by increasing the turnover of antioxidant enzymes, such as glutathione peroxidase, catalase, superoxide dismutase (Jomova et al., 2023). Studies using HT-1080 cell cultures (fibrosarcoma cell line) showed that the addition of catalase to cell culture decreases hydrogen peroxide production after 3 and 24 h of oxygen deprivation and that this protection is dose-dependent (Zitta et al., 2010). In our study, Brosimine B could counteract oxidative stress only up to 3 h of OGD (see Figure 5). Since Brosimine B effectively increased cell viability after this period, it may be supporting other neuroprotective mechanisms.

Besides demonstrating the potential of Brosimine B as a neuroprotective agent, our results also highlight its hormetic behavior in retinal cell cultures. The biphasic dose-response was effectively modeled using an inverted U-shaped hormetic function, confirming the biphasic nature of Brosimine B’s dose-response and reinforcing its hormetic behavior, also observed in other natural compounds (Mattson, 2008; Hajialyani et al., 2019). Specifically, our model suggests that Brosimine B promotes cell viability at low concentrations (∼10 µM) but becomes cytotoxic at higher doses, revealing a peak viability at ∼10.2 µM and a spread (σ = 6.5 µM) that delineates the hormetic zone. From a pharmacological perspective, the hormetic model aligns well with established dose-response patterns of neuroprotective flavonoids, such as hesperidin (Chang et al., 2015) and acetonide (Xia et al., 2018). To further refine these parameters and validate the therapeutic index, additional studies should incorporate more data points within the 5–25 µM range, offering a more detailed view of the hermetic window for Brosimine B.

In future studies, we also intend to compare the neuroprotective effects of Brosimine B in OGD cultures against positive controls, such as other available drugs with confirmed neuroprotective effects against brain ischemia. Another future goal is to chart the cell signaling pathways underlying the neuroprotective effects of Brosimine B.

## 5 Conclusion

Our results indicate that the compound 4′,7-dihydroxy-8-(3,3-dimethylallyl)flavan (Brosimine B) protects chicken embryo retinal cells under *in vitro* hypoxic conditions (OGD), likely due to its intrinsic antioxidant properties and its ability to enhance endogenous cellular antioxidant defenses. These findings provide valuable insights about Brosimine B properties, mechanism of action, potential therapeutic effects, and hormetic behavior, which can inform and guide the design of subsequent *in vivo* studies aiming to rescue nerve cells from hypoxia, such as those located in the penumbra region after stroke.

## Data Availability

The raw data supporting the conclusions of this article will be made available by the authors, without undue reservation.
