# Supplementary material for: Brosimine B and the biphasic dose-response: insights into hormesis and retinal neuroprotection
Source: Front Pharmacol. 2025 Apr 8;16:1558726. doi: 10.3389/fphar.2025.1558726 (PMC12012618; doi:10.3389/fphar.2025.1558726)
Supplement: Supplementary file 1 [file DataSheet1.docx]

***Supplementary Material***

**Brosimine B and the Biphasic Dose-Response: Insights into Hormesis and Retinal Neuroprotection.**

**Susanne Suely Santos da Fonseca^1,6*^, Natacha M. de S. Port’s^3^, Gisele Priscila Soares de Aguiar^3^, Eliã P. Botelho^3^, Nádia M. G. de Couto^2^, Wandson Braamcamp de Souza Pinheiro^2^, André Salim Khayat^6^, Elizabeth S. Yamada^4^, Edmar T. da Costa^4^, Chubert Bernardo C. de Sena^5^, Mara Silvia P. Arruda^2^, Carlomagno P. Bahia^3^, Antonio Pereira Junior^1^**

*** Correspondence:**Susanne Suely Santos da Fonseca

[susannesantos@outlook.com](mailto:susannesantos@outlook.com)

# Supplementary Figures


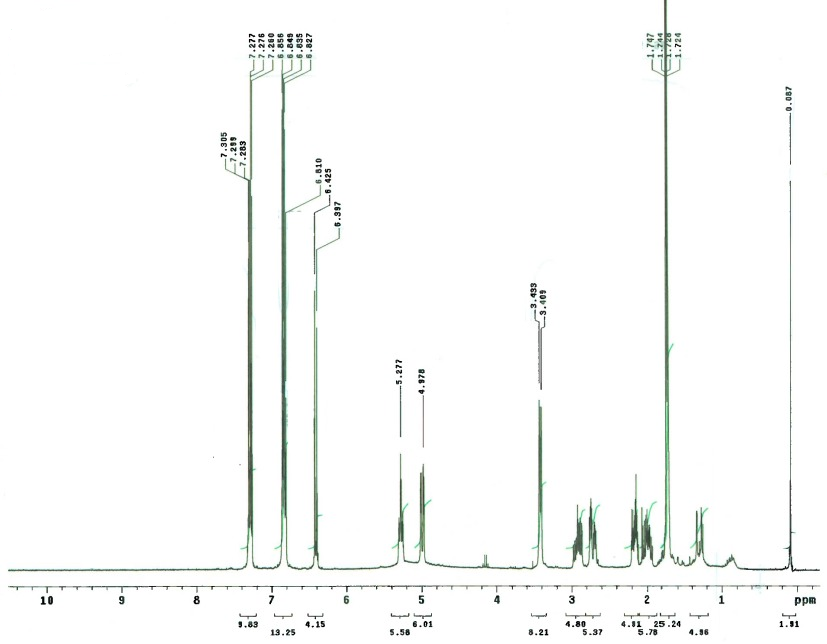


**Supplementary Figure 1.** 1H NMR spectrum (300 MHz, CDCl3) of 4,7-dihydroxy-8-(3,3-dimethylallyl)flavan (Brosimine B).

**
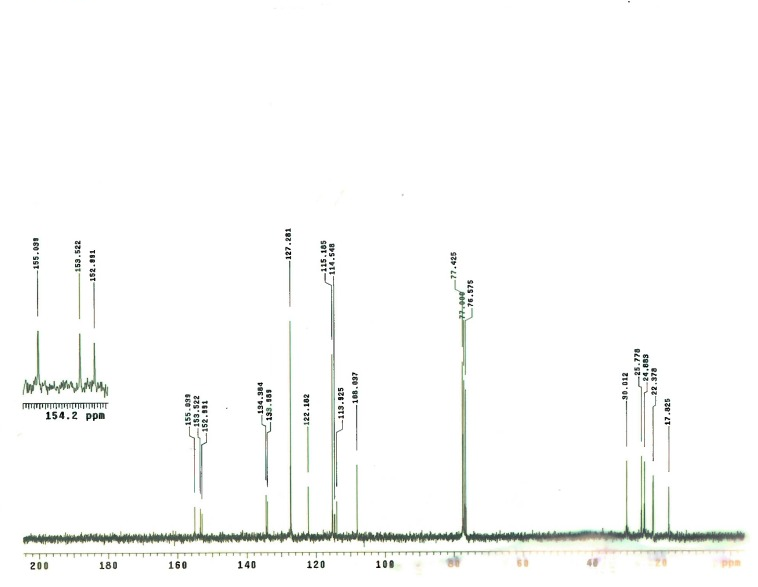
**

**Supplementary Figure 2.** 13C NMR spectrum of 4,7-dihydroxy-8-(3,3-dimethylallyl)flavan (Brosimine B).
